# A Rare Case of an Irreducible Inguinoscrotal Hernia (Scrotal Cystocele) With a Left Vaginal Hydrocele: A Case Report

**DOI:** 10.7759/cureus.69413

**Published:** 2024-09-14

**Authors:** Pooja Chandak, Abhishek Ingole, Kanhaiya B Chandak

**Affiliations:** 1 Medicine and Surgery, Jawaharlal Nehru Medical College, Datta Meghe Institute of Higher Education and Research, Wardha, IND; 2 Community Medicine, Jawaharlal Nehru Medical College, Datta Meghe Institute of Higher Education and Research, Wardha, IND; 3 Surgery, Lotus Hospital and Research Centre, Nagpur, IND

**Keywords:** cect, hydrocele, inguinal bladder hernia, open surgery, scrotal cystocele

## Abstract

Inguinal bladder hernia, also known as scrotal cystocele, first described by Dr. Levine, is considered among the rare cases that may or may not present as scrotal swelling depending on the size of the herniated part. In this particular patient, he presented with scrotal swelling and symptoms related to difficulties in micturition, such as incomplete voiding and increased frequency of voiding urine. On contrast-enhanced computed tomography (CECT), it was revealed that the bladder had been herniated into the inguinoscrotal region, accompanied by a left vaginal hydrocele. The hydrocele was then drained, and the left scrotal sac was everted. The hernial contents were reduced, and an open mesh repair was done to reduce the incidences of recurrence. These cases can generally be seen in obese male patients who are more than 50 years of age and males above 50 years with associated comorbidities. The diagnosis plays a very crucial role in these cases; if the presence of the bladder is not diagnosed preoperatively, then there is a fair chance that the bladder can undergo iatrogenic trauma during surgery. Hence, the clinician should be aware and should consider this situation if the patient comes under the criteria of being above 50 years old, with obesity and/or comorbidities, and with/without symptoms. Although rare, the condition can be treated by undergoing an open surgical repair, wherein the hernial contents are reduced and mesh is put in to avoid reoccurrence.

## Introduction

Dr. David B. Levine was the first to describe inguinal bladder hernia in 1951, also known as "scrotal cystocele" [1]. It is considered a rare clinical condition with only around 1%-3% incidence, although it can range up to 10% in obese men with an age greater than 50 years [2]. Any segment of the bladder, ranging from the diverticulum to the entire bladder itself, can be herniated into the inguinoscrotal sac [3,4]. Generally, these types of hernias are predominant on the right side [5]. Typically, the diagnosis is established during surgery, as these patients rarely exhibit any symptoms. However, approximately 7% of patients are diagnosed preoperatively due to symptoms such as difficulty in voiding urine, increased frequency of micturition, and other urological complaints, which avoids iatrogenic trauma to the urinary bladder [6,7]. Generally, open repair is the procedure of choice for this kind of condition [4]. We present a case of an irreducible left inguinoscrotal hernia with the urinary bladder as the content (scrotal cystocele) along with a left vaginal hydrocele.

## Case presentation

A 75-year-old Indian male presented to the surgery OPD with chief complaints of irreducible swelling over the left inguinoscrotal region for the last two months. He also complained of increased frequency of micturition and an incomplete sense of micturition, along with occasional mild pain while voiding urine. There was no history of abdominal distention, abdominal pain, or any bowel complaints. The patient passes flatus and stools regularly without any complaints of constipation. The patient also complained of a reducible swelling in the inguinal region for five years. The patient has a known case of hypertension and also had a history of ischemic heart disease (IHD), having undergone coronary artery bypass graft (CABG) surgery eight years ago, and transurethral resection of the prostate (TURP) six years ago.

On general examination, vitals were stable, and the patient was conscious and well-oriented to time, place, and person. Bilateral air entry was present, suggesting normal functioning of the respiratory system. S1 and S2 were heard normally without any murmurs. The abdomen was soft and non-tender. On local examination, the swelling on the left side was non-reducible, and no cough impulse was present. The swelling was soft and cystic, along with a positive fluctuation test. Transillumination was negative. Testis is not separately felt. The right inguinoscrotal region was apparently normal on local examination. Reports of the basic investigations, including 2D echo and ECG, were normal. The patient was sent for an ultrasonography of the scrotum, which suggested a prostatomegaly along with a fluid collection of approximately 30 cc in the left inguinoscrotal region, favoring a left vaginal hydrocele. No bowel loop herniation into the inguinal region was seen.

**Figure 1:**
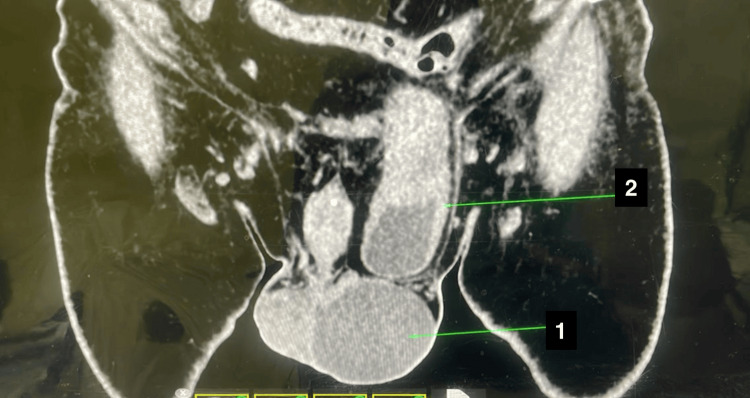
CT reports showing a hydrocele and herniated urinary bladder in coronal view; arrow 1 indicates a hydrocele sac; arrow 2 indicates an irreducible urinary bladder

**Figure 2:**
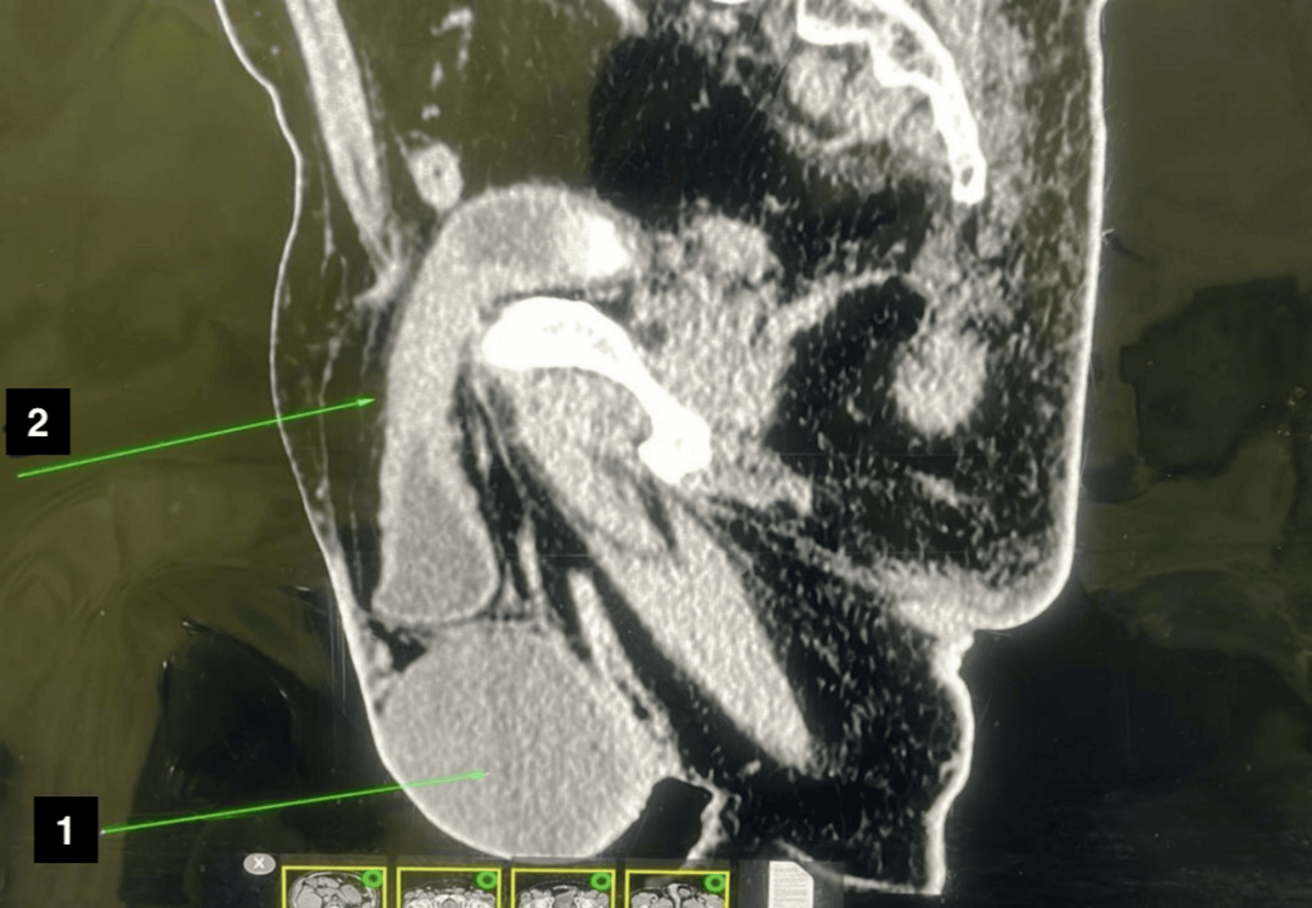
CT report showing a herniated urinary bladder and hydrocele in sagittal view; arrow 1 indicates a hydrocele sac; arrow 2 indicates an irreducible urinary bladder

Due to the strong clinical history of reducible swelling in the inguinal region, the patient was further evaluated on contrast-enhanced computed tomography (CECT). The findings indicated the presence of an approximately 11 cm long blindly ending outpouching, measuring approximately 1.5 cm at its origin. This outpouching was observed to arise from the anterolateral aspect of the left lateral wall of the urinary bladder, which exhibited mildly enhanced symmetrical wall thickening, with a maximum wall thickness of approximately 6 mm. It was seen to be inferiorly herniating into the left inguinoscrotal region. Prostatomegaly was also noted, with prostate size measuring approximately 5.1 × 4.2 cm in size in the axial plane. No bowel loop herniation was seen on either side of the inguinal canal. No evidence of intravesical mass or calculus was seen. The clinical examination and CECT scan report led to the diagnosis of an irreducible left inguinoscrotal hernia, with the urinary bladder identified as the content (scrotal cystocele), along with a left vaginal hydrocele (Figures 1, 2).

**Figure 3:**
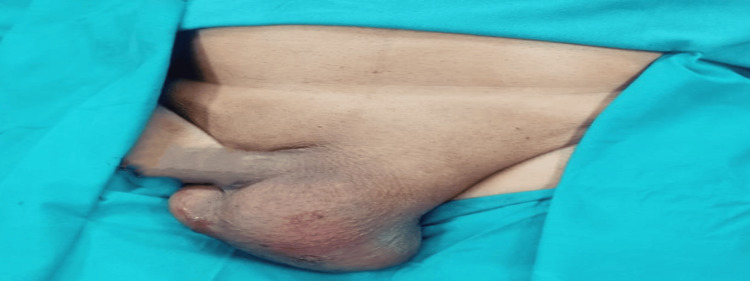
Preoperative image of the operative site

Upon admission, following the physician and anesthetist assessments (Figure 3), the patient was posted for surgery.

Intraoperatively, the findings of the CECT scan were confirmed, hernial contents were reduced, and the left hydrocele was drained, followed by the eversion of the sac. The patient underwent an open mesh repair through the Lichtenstein technique. The post-op period was uneventful as the patient was started on an oral diet. Six hours after the surgery, urinary symptoms improved, and he was discharged on day three of the operation. The patient was doing well during the follow-up visit when stitches were removed, and no significant event occurred.

## Discussion

Hydrocele and inguinal hernias are among the most common case scenarios encountered by general surgeons. The omentum and small intestines are the most frequently encountered contents of a hernia; however, there are rare instances where the herniated contents may include the appendix, colon, ovary, fallopian tubes, bladder, etc. [1]. Hydrocele is a prevalent condition that can be categorized into two main types: primary hydrocele and secondary hydrocele [8]. In this case, the unilateral presentation of the urinary bladder as the content of the hernial sac on the left side is an unusual event in itself because, generally, right-sided herniation of the urinary bladder is more common [4]. The presentation is also accompanied by left-sided vaginal hydrocele, which makes the condition even more rare as only about 1%-3% of inguinal hernia cases are of urinary bladder hernia [2]. Based on the relation between the peritoneum and hernia, they are classified into three types: paraperitoneal, intraperitoneal, and extraperitoneal [1].

Patients beyond the fifth decade, along with symptoms such as bladder outlet obstruction (BOO), weak abdominopelvic wall, direct inguinal hernia, weak bladder tone, obesity, and male gender as the pathophysiological factors, are more susceptible to this condition compared to others [7]. In this case, the patient has undergone TURP, suggestive of BOO and weak pelvic muscles, which acts as a potential factor for herniation of the urinary bladder. Inguinal bladder hernias are mostly asymptomatic, especially where only a small portion of the bladder is herniated, implying that the diagnosis in these cases is generally done intraoperatively. However, in hernias where a large portion of the bladder has been herniated, the patient invariably presents with a swelling in the groin and/or scrotal region and lower urinary tract symptoms [4]. These symptoms may be nonspecific, such as hesitancy in urination, increased frequency of voiding, burning sensation, hematuria, dysuria, and frequent urinary infections that can be associated with BOO [1,9].

The majority of inguinal bladder hernias are diagnosed intraoperatively, and approximately 7% of these cases are diagnosed preoperatively through various diagnostic modalities such as ultrasonography, intravenous pyelography, cystography, and CT scans [1,4]. In this case, the ultrasonography did not point toward any bladder involvement but implied a left vaginal hydrocele. However, since the patient complained of reducible swelling in the inguinal region for the last five years, along with the increased frequency of micturition and incomplete voiding of urine, the patient was further investigated on a CECT scan, which showed the herniation of the bladder in the inguinal region, confirming the clinical suspicion and avoiding iatrogenic trauma to the urinary bladder.

The recommended approach in these situations is open surgical repair, which involves reducing the hernial contents and placing a mesh utilizing the Lichtenstein technique [1,4]. A part of the bladder is only resected if findings of necrosis are indicated [1]. In this case, a left vaginal hydrocele was also present, leading to the drainage of the left hydrocele and the eversion of the sac.

## Conclusions

An inguinal bladder hernia represents an unusual clinical manifestation, and this specific case is considered rare due to the concurrent presence of the inguinal bladder hernia along with a left vaginal hydrocele. In such cases, if the bladder is not identified prior to surgery, there exists a risk of iatrogenic injury to the bladder; therefore, comprehensive investigations by the clinician are recommended. The clinical scenario can be detected using various investigation modalities such as ultrasonography and CT scans, along with close observation of the patient's symptoms if they are present, such as increased frequency of micturition, incomplete voiding of urine, and burning sensation, as these patients are largely asymptomatic. The preferred mode of treatment in such cases is the open surgical approach.
